# Conducting evidence synthesis and developing evidence-based advice in public health and beyond: A scoping review and map of methods guidance

**DOI:** 10.1017/rsm.2025.10051

**Published:** 2025-11-18

**Authors:** Ani Movsisyan, Kolahta Asres Ioab, Jan William Himmels, Gina Loretta Bantle, Andreea Dobrescu, Signe Flottorp, Frode Forland, Arianna Gadinger, Christina Koscher-Kien, Irma Klerings, Joerg J. Meerpohl, Barbara Nussbaumer-Streit, Brigitte Strahwald, Eva A. Rehfuess

**Affiliations:** 1 Institute for Medical Information Processing, Biometry and Epidemiology (IBE), Chair of Public Health and Health Services Research, Faculty of Medicine, LMU Munich, Germany; 2 Norwegian Institute of Public Health, Norway; 3 Institute for Evidence in Medicine, Medical Centre, Faculty of Medicine, University of Freiburg, Germany; 4 Department for Evidence-based Medicine and Evaluation, University for Continuing Education Krems, Austria

**Keywords:** evidence-based advice, evidence-informed decision-making, evidence synthesis, methods guidance, public health

## Abstract

Effective public health decision-making relies on rigorous evidence synthesis and transparent processes to facilitate its use. However, existing methods guidance has primarily been developed within clinical medicine and may not sufficiently address the complexities of public health, such as population-level considerations, multiple evidence streams, and time-sensitive decision-making. This work contributes to the European Centre for Disease Prevention and Control initiative on methods guidance development for evidence synthesis and evidence-based public health advice by systematically identifying and mapping guidance from health and health-related disciplines.

Structured searches were conducted across multiple scientific databases and websites of key institutions, followed by screening and data coding. Of the 17,386 records identified, 247 documents were classified as ‘guidance products’ providing a set of principles or recommendations on the overall process of developing evidence synthesis and evidence-based advice. While many were classified as ‘generic’ in scope, a majority originated from clinical medicine and focused on systematic reviews of intervention effects. Only 41 documents explicitly addressed public health. Key gaps included approaches for rapid evidence synthesis and decision-making and methods for synthesising evidence from laboratory research, disease burden, and prevalence studies.

The findings highlight a need for methodological development that aligns with the realities of public health practice, particularly in emergency contexts. This review provides a key repository for methodologists, researchers, and decision-makers in public health, as well as clinical medicine and health care in Europe and worldwide, supporting the evolution of more inclusive and adaptable approaches to public health evidence synthesis and decision-making.

## Highlights

### What is already known?

- Methods guidance for evidence synthesis and evidence-based advice has been predominantly developed for clinical medicine, focusing on intervention effectiveness and structured decision-making processes.

### What is new?

- This scoping review systematically maps existing methods guidance across a range of research questions and types of scientific enquiry and identifies key gaps in the applicability of methods guidance to public health, particularly in rapid response, integration of diverse evidence types, and real-world decision-making contexts.

### Potential impact for RSM readers

- The findings offer both a repository for those conducting evidence synthesis and developing evidence-based advice and a roadmap for those seeking to advance methodological approaches that better align with public health needs. They support the development of adaptable and broader approaches to evidence synthesis and evidence-based advice in public health. The interactive evidence map is available here: https://ew6.ibe.med.uni-muenchen.de/mediate/evidence-gap-map/.

## 1 Introduction

Effective public health decision-making depends on timely and trustworthy evidence. Evidence synthesis plays a critical role in developing evidence-informed recommendations by systematically collecting, appraising, and synthesising available research on a range of different questions. To support this process, methods guidance on how to conduct evidence synthesis and formulate evidence-based advice—defined broadly as public health recommendations or guidance derived from a systematic consideration of the available evidence—is crucial in ensuring quality, consistency, and transparency across public health recommendations.^1^ However, most existing methods guidance has been developed in clinical medicine under the evidence-based medicine paradigm, primarily focusing on questions of intervention effectiveness and safety (i.e., benefit-harm balance) and selected methodologies (e.g., randomised controlled trials).^2^

Over time, the principles of evidence-based decision-making have expanded beyond clinical medicine to other fields, including public health, where demands tend to be more complex. While key principles still apply, public health decision-making addresses population-level health impacts with equity being a core value. It often concerns sectors beyond the health sector, where various consequences beyond those intended, as well as acceptability, feasibility, and other implementation considerations, play a critical role.^3^^–^ ^5^ Public health measures tackle multiple health determinants (e.g., social, environmental, and behavioural),^6^ interact with diverse socio-economic and political factors, and require integration of multiple streams of evidence to address a wide range of questions.^4^ These complexities call for tailored methods guidance to ensure appropriate and actionable evidence-based advice for public health.

The need for reliable, transparent, and systematic approaches becomes even more acute during public health emergencies.^7^^,^ ^8^ In such situations, decision-makers must act swiftly under dynamically evolving conditions and in a climate of much uncertainty, relying on incomplete and rapidly evolving evidence. The COVID-19 pandemic highlighted critical gaps in existing methods guidance, which often proved inadequate, notably with regards to rapid approaches in the face of pressing decision-making needs.^9^ Public health agencies, both nationally and internationally, faced the dual challenge of having to inform decisions with far-reaching societal implications and of having to build on a rapidly changing and often very limited evidence base.^10^

In response to these challenges and informed by lessons from the COVID-19 pandemic,^4^ the European Centre for Disease Prevention and Control (ECDC)—the European Union (EU)‘s public health agency—has expanded its mandate to include the provision of evidence-based recommendations to guide infectious disease prevention and control.^11^^,^ ^12^ Previously, ECDC’s primary role was to collect and analyse data on infectious diseases, such as COVID-19, influenza, and tuberculosis, as well as public health issues, such as antimicrobial resistance and vaccination. While it has long played a critical role in assessing health risks for countries in the EU and the European Economic Area, its post-pandemic role now also explicitly includes evidence-based advice.^12^ To support these responsibilities, ECDC launched an initiative that aims to develop practical, fit-for-purpose methods guidance for evidence synthesis and evidence-based public health advice, focusing on infectious disease epidemiology, prevention, and control.

In the context of this initiative, the MEthoDs guidance for public heAlTh Evidence synthesis and evidence-based advice (MEDIATE) consortium was commissioned to take stock of existing methods guidance.^13^ The present scoping review systematically identifies and maps existing methods guidance on evidence synthesis and evidence-based advice, with the aim to chart the existing landscape and highlight areas where further development could be beneficial. The dual framing of this work—as a scoping review and an evidence map—served two complementary functions. The scoping review methodology enabled a comprehensive and systematic identification of relevant guidance, while the evidence mapping approach provided a structured framework for organising and visualising key characteristics of the guidance and the existing gaps. By focusing on both routine and emergency contexts, the review provides an important resource for everyone producing evidence synthesis and evidence-based advice in the health field and for methodologists seeking to advance methods for evidence-based public health decision-making.

## 2 Methods

The protocol for this review was registered a priori with the Open Science Framework and can be found at https://doi.org/10.17605/OSF.IO/WMXCN. Key methodological decisions are summarised in the following sections and reported in line with the Preferred Reporting Items for Systematic Reviews and Meta-Analyses (PRISMA) extension for scoping reviews.^14^

### 2.1 Criteria for including methods guidance

We defined methods guidance as documents that provide a set of principles, structured processes, recommendations, or the like regarding the conduct of evidence synthesis or the development of evidence-based advice. Importantly, such guidance had to address the overall process of developing evidence synthesis or evidence-based advice rather than address specific steps only (e.g., methods guidance focusing only on critical appraisal of studies). We did not apply restrictions based on publication type. The methods guidance could have been developed and published by researchers, such as in peer-reviewed journals, or developed and/or used by key institutions in health care. Key institutions were defined as entities that develop and disseminate methods guidance for evidence synthesis or guideline development, promoting research transparency and quality (e.g., Cochrane Collaboration, Joanna Briggs Institute [JBI]), or as global, national, and regional health organisations that commission or conduct evidence synthesis and evidence-based advice (e.g., WHO, national public health institutes). The MEDIATE consortium collaboratively identified 26 key institutions (see File S1 of the Supplementary Material).

To be included, methods guidance had to focus on any type of evidence synthesis (e.g., systematic reviews, scoping reviews, overviews of reviews) and/or any type of evidence-based advice (e.g., evidence-based guidelines or recommendations and health technology assessments (HTAs)) directed at populations/groups rather than individuals/patients. Given the lack of a classification of types of evidence-based advice, our approach was inclusive, considering methods guidance for any type of advice that is the result of a structured and transparent development approach using the best available evidence and expert assessment to varying extents. We excluded methods guidance solely concerned with knowledge dissemination, knowledge translation, or science communication, as well as guidance focusing on reporting standards. While we recognise that EtD frameworks may be viewed as a tool to facilitate knowledge translation, EtD frameworks are not primarily designed or exclusively used for this purpose. They therefore fit this review’s focus on guidance that informs the full process of evidence synthesis or structured formulation of advice, rather than guidance on dissemination or implementation.

We included guidance published from 2000 onwards. This cut-off choice is rooted in the historical progression of evidence-based methodologies. While the term ‘evidence-based medicine’ was introduced in the 1990s, marking the start of a period of foundational growth and development in relevant methods, it was the early 2000s that saw the publication of key standards and methodological benchmarks, such as PRISMA^15^ and AGREE (Appraisal of Guidelines for Research & Evaluation).^16^ By focusing on literature published during the last 20+ years, our intention was to capture the refinement and expansion of evidence-based methodologies that followed these important advancements and ensure our focus remains on the most relevant and contemporary methodological approaches. In line with our review protocol, we included guidance applicable across any health discipline (e.g., clinical medicine, public health) and concerning any health issue (e.g., infectious diseases, non-communicable diseases). While our primary interest in this map was public health, we adopted broader inclusion criteria encompassing multiple disciplines to allow capturing evidence-based principles that are foundational across disciplines and hence also applicable to public health. Public health often intersects with the social sciences—particularly in areas such as health systems, policy, and community interventions—so we included guidance from organisations, such as the Campbell Collaboration, when it was concerned with methods directly applicable to evidence synthesis or advice generation in health/wellbeing-related contexts. However, we did not include guidance focused solely on non-health disciplines such as education or criminal justice.

### 2.2 Search methods

We used a multi-pronged approach to searching that combines: (i) systematic searches in scientific databases, (ii) searches on the websites of key institutions, and (iii) citation searches based on identified relevant documents (i.e., network searches of related articles). We conducted searches in English but included methods guidance published in all other languages.

#### 2.2.1 Systematic searches in scientific databases

The searches were designed by an experienced information specialist (IK). They were developed using both free text and relevant controlled vocabulary terms, if available. The following databases were searched on 24 April 2024: Ovid Medline ALL, Web of Science Core Collection (Science Citation Index Expanded (SCI-EXPANDED), Social Sciences Citation Index (SSCI), Arts & Humanities Citation Index (AHCI), Emerging Sources Citation Index (ESCI))00), and the Library of Guidance for Health Scientists (LIGHTS) (https://lights.science/). The Medline search strategy was assessed by a second information specialist using the Peer Review of Electronic Search Strategies (PRESS) guideline.^17^ The search blocks focused on (i) types of evidence synthesis and evidence-based advice (e.g., systematic reviews, scoping reviews, HTAs), (ii) conduct (e.g., development, reporting), and (iii) guidance (e.g., manual, guidance, guideline). We searched LIGHTS using the filters for systematic reviews, HTAs, meta-analyses, and clinical practice guidelines to identify relevant guidance. Because the database currently has no export function, the LIGHTS team provided us with a list of PMIDs and DOIs to enable the downloading of references via citationchaser (https://estech.shinyapps.io/citationchaser/). The full search strategy is available in File S2 of the Supplementary Material.

All search results were deduplicated using EPPI Reviewer 6.

#### 2.2.2 Searches on the websites of key institutions

An information specialist advised on the comprehensive search strategy for key institutions’ websites. For a systematic, transparent, and reproducible approach, we used the following search approaches on the websites of the key institutions:

1. *Manual website navigation*: Websites were explored using their built-in structure, navigating sections, such as publications, resources, and libraries.
2. *Keyword searches:* Search terms like ‘handbook’ ‘manual’ ‘guidance’ ‘systematic review’ and ‘evidence synthesis’ and their combinations were systematically applied using each website’s search function.
3. *Google-limited searches*: For websites without functional search tools, we conducted Google site-limited searches using predefined combinations of keywords (e.g., *site:cochrane.org handbook OR guidance ‘systematic reviews’*).

The first 20 results from each query were screened for relevance. All search activities were documented, including the search date, source, method, and number of retrieved documents. This approach was tailored to the structure and functionality of each institution’s website.

#### 2.2.3 Network searches

We ran a network search on 2 June 2024 on 247 guidance documents identified during full text screening in OpenAlex (https://openalex.org/)^18^ using EPPI Reviewer 6^19^ based on high relevance guidance (see further details under Results below) identified from our database searches and full text screening (applying ‘bibliography’ and ‘recommended by’ functions).

### 2.3 Selection of methods guidance

We first screened the titles and abstracts of the identified records using EPPI Reviewer.^19^ All reviewers involved with this stage of screening piloted the screening guidance by screening the same 50 records. Based on this, we revised and standardised the screening guidance. Eight reviewers (AD, AG, AM, BNS, CK, GB, JH, KAI) independently screened the titles and abstracts using a conservative approach, meaning if there were any uncertainties regarding the inclusion of a record, that record was moved to full-text screening without a second review. We used priority screening for this stage, a method that ranks studies based on a machine learning (ML) model to prioritise the studies that are most likely to be relevant, ensuring that the records most likely to be included are screened first. As we approached the threshold beyond which no further relevant records were expected, we screened an additional 300 lower-ranked records to validate this. Since none met the inclusion criteria, we concluded screening and relied on the ML model’s prioritisation.

The same process was used for full text screening. Seven reviewers (AD, AM, BNS, CK, GB, JH, and KAI) independently screened the full texts. If there were uncertainties regarding the inclusion of a record, a second reviewer reviewed the record before a final decision on inclusion or exclusion was made.

### 2.4 Data extraction and development of the initial map of methods guidance

We developed an a priori mapping framework for categorising identified methods guidance. This was drafted by AM and ER, refined through iterative discussions within the team and tested on selected methods guidance documents (e.g., Cochrane Handbook) to ensure the framework’s depth and practicality. While according to our protocol (https://doi.org/10.17605/OSF.IO/WMXCN), we had initially planned to conduct more detailed data mapping, extracting and summarising textual data on reported experiences with the methods guidance and their limitations/gaps, we did not proceed for feasibility reasons, given the very large number of methods guidance identified. The domains of the final mapping framework are presented in Table 1.

**Table 1:** Domains of the mapping framework

Type of guidance	*Evidence synthesis* Systematic review of quantitative data Scoping review Evidence (gap) map Overview of reviews/umbrella reviews Qualitative evidence synthesis Mixed-methods evidence synthesis Other *Evidence-based advice* Guidelines/recommendations Evidence-to-decision considerations Health technology assessment (HTA) Other
Type of question	Prevalence/incidence Disease burden Clinical manifestation Aetiology/risk factors Prognosis Diagnostics Laboratory-based aspects Interventions Implementation Economic considerations Other
Production mode	Normal (i.e., production within standard timelines including a few months) Rapid (within a few weeks) Ultra-rapid (within 48 hours) Living

Additionally, we coded the specific *health area* covered (i.e., infectious diseases, non-communicable diseases, and injuries), the *focus area* (i.e., clinical, public health, and service delivery), and marked any *cross-cutting themes* that emerged (e.g., how to incorporate equity considerations, complexity, stakeholder engagement, and automation/ML).

The map was developed using the EPPI mapper, a web-based tool designed to create interactive visualisations for organising and exploring evidence.^20^ This interactive map allows users to navigate the methods guidance by applying the pre-defined categories in a dynamic format. One reviewer first mapped 50 methods guidance documents based on the a priori framework, validating the application of the mapping framework in consultation with the other reviewers. Seven reviewers (AD, AM, BNS, CK, GB, JH, and KAI) then coded the remaining methods guidance documents using the pre-defined mapping categories. Senior researchers in the team provided feedback on the mapping process and presentation of the findings in the initial map (ER, FF, SAF, and JM).

### 2.5 External consultations and map refinement

To refine the initial map of methods guidance, we conducted a brief survey with 53 methodologists from across the world identified through the project partners’ network, receiving 13 responses (25% response rate). Participants reviewed the map and provided general feedback. They were also specifically asked to suggest additional guidance, particularly for rapid (i.e., production within a few weeks) and ultra-rapid approaches (i.e., production within a few days), as well as provide information on ongoing projects to develop methods guidance. The survey, conducted via LimeSurvey^21^ from July 27 to August 30, 2024, included a link to the initial map for review.

Additionally, the initial map was shared with 18 members of ECDC’s Methodological Advisory Group (MAG), convened by the ECDC Chief Scientist to advise the agency on how to strengthen evidence-based approaches. This is composed of methodologists and representatives of European national public health institutes. Four MAG members (22% response rate) provided feedback.

All input from methodologists and MAG members was synthesised to refine and update the map. Respondents consenting to be named are listed in File S3 of the Supplementary Material.

## 3 Results

### 3.1 Results of the search

**Figure 1:**
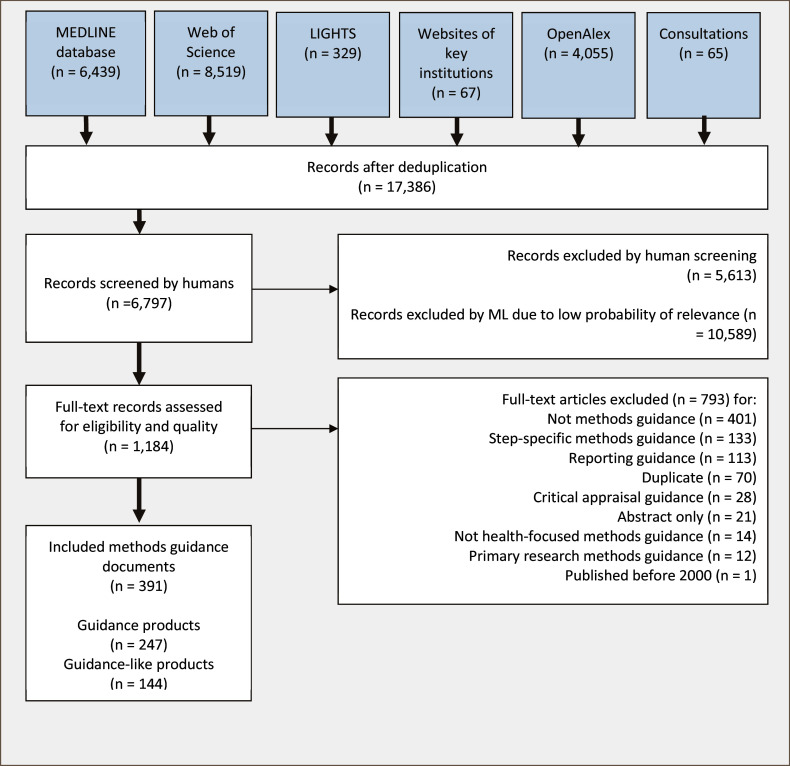
PRISMA flow diagram. *Although we conducted deduplication in EPPI Reviewer prior to the title and abstract screening, a few duplicates were still identified during the screening process. These duplicates were due to inconsistencies in the reporting of titles, issues, and volumes, which were not detected by EPPI Reviewer.

Figure 1 provides a graphical representation of our search and guidance selection results. In total, we identified 17,386 unique records through systematic searches in databases, key institutions’ websites, and network searches, as well as through consultations with methodologists and MAG members. These consultations yielded a total of 65 records, of which 58 were unique and not found through literature searches.

Out of the 17,386 unique records, we manually screened 6,797 (including the 58 records suggested by methodologists and MAG members) and excluded 10,589 based on a low likelihood of relevance, as indicated by the ML model. This model was trained to differentiate between manually identified included records and excluded records. As we reached the threshold where we did not expect further relevant includes, we continued to screen an additional 300 records to ensure the validity of the threshold. As none of these 300 records were found to be eligible, we felt safe to stop screening and rely on the ML model. The 30% likely relevance threshold, which was chosen based on evaluations and experience at the Norwegian Institute of Public Health,^22^ was found appropriate as no further relevant methods guidance was identified.

In total, we screened 1,184 full texts, of which 391 met our inclusion criteria. Among these, 247 records were classified as guidance products. The remaining 144 records were classified as guidance-like products; while not explicitly labelled as guidance, these documents provided broader literature reviews and often overviews of existing guidance detailing a step-by-step approach to evidence synthesis or evidence-based advice. We decided to include them in the map, adding an additional code to differentiate between guidance and guidance-like products. Filtering on this code allows users to decide for themselves whether they consider them relevant or not. However, for the network searches, we used only the guidance products.

### 3.2 Development of the map

As described above, we created an initial map using only records identified through literature searches. We refined the initial map based on feedback from external methodologists and MAG members. Following the consultations, we made the following adaptations:

- We added 18 additional guidance documents to the map from the 58 unique records suggested by methodologists and MAG members. Of these, 13 were classified as guidance products, and five as guidance-like products.
- We reclassified selected guidance products based on specific suggestions. For instance, with regards to ‘type of question’, methods guidance on qualitative evidence synthesis was reassigned from the category ‘intervention’ to ‘other’, as it was found to address multiple broader questions related to experiences, values, and preferences.
- For guidance products concerned with evidence-based advice, we omitted the specification of ‘type of question’. These products primarily address management (i.e., advice on how to intervene), rendering many of the predefined question types (e.g., prevalence/incidence, disease burden) irrelevant.
- We updated the references for some guidance products with the latest versions available. In cases where the previous versions of the guidance were available online (e.g., on the websites of the key institution), these were included and counted as distinct guidance products.

These consultations also revealed 13 ongoing projects on the development of methods guidance; however, none had a completed guidance product at the time of the map’s finalisation.

### 3.3 Characteristics of included guidance products

Table 2 provides an overview of the characteristics of the 247 included guidance products.

**Table 2:** Characteristics of included methods guidance products

	Guidance (*n* = 247)
**Publication year**	
2000–2005	12 (5%)
2006–2010	19 (8%)
2011–2015	57 (23%)
2016–2020	77 (31%)
2021–2024	79 (32%)
Publication year not reported	3 (1%)
**Type of guidance** ^**1**^	
**Evidence synthesis**	**155**
Systematic review of quantitative data	116 (75%)
Scoping review	9 (6%)
Evidence (gap) map	1 (0.6%)
Overview of reviews/umbrella review	8 (5%)
Qualitative evidence synthesis	19 (12%)
Mixed-methods evidence synthesis	5 (3%)
Other	4 (3%)
**Evidence-based advice**	**98**
Health technology assessment	11 (11%)
Guidelines/recommendations	77 (79%)
Evidence-to-decision considerations	10 (10%)
Other	6 (6%)
**Health area**	
Infectious diseases	12 (5%)
Non-communicable diseases	23 (9%)
Injuries	3 (1%)
Generic	212 (86%)
**Focus area**	
Clinical	80 (32%)
Public health	41 (17%)
Service delivery	12 (5%)
Generic	138 (56%)
**Cross-cutting themes addressed**	
Equity	19 (8%)
Complexity	17 (7%)
Stakeholder engagement/participation	13 (5%)
Adaptation	5 (2%)
Automation/machine learning	4 (2%)
Context & implementation	16 (6%)
Ethics	8 (3%)
**Production mode**	
Normal	215 (87%)
Rapid	19 (8%)
Ultra-rapid	0
Living	15 (6%)

^1^Some guidance products fall into more than one sub-category, therefore the percentages in each category do not add up to 100%.

Most of the included methods guidance products were published from 2011 onwards (216 guidance products, 87%), with the highest concentration between 2021 and 2024 (79 products, 32%).

The included methods guidance more often addressed the development of evidence synthesis (155 products, 63%) than evidence-based advice (98 products, 40%), with some guidance covering both areas. In evidence synthesis, most focused on systematic reviews of quantitative data (116 products, 75%); in evidence-based advice, most focused on guidelines/recommendations (77 products, 79%).

In terms of specific health areas, most guidance was classified to be generic, meaning they did not specify a health area (212 products, 86%); we found only 12 guidance products 5% addressing infectious diseases.^23^^–^ ^34^ Similarly, while most guidance products did not specify a focus area, a large proportion had a clinical focus (80 products, 32%) with only 41 products (17%) classified as addressing public health.

Sixty of the included methods guidance products (24%) addressed cross-cutting themes: equity (19 products, 8%),^3^^,^ ^27^^,^ ^31^^,^ ^34^^–^ ^49^ complexity (17 products, 7%),^3^^,^ ^34^^,^ ^38^^,^ ^45^^,^ ^49^^–^ ^61^ context and implementation (16 products, 6%),^47^^,^ ^49^^,^ ^62^^–^ ^75^ stakeholder engagement (13 products, 5%),^29^^,^ ^31^^,^ ^37^^,^ ^38^^,^ ^46^^,^ ^49^^,^ ^76^^–^ ^82^ ethics (8 products, 3%),^34^^,^ ^44^^,^ ^49^^,^ ^69^^,^ ^83^^–^ ^86^ adaptation (5 products, 2%),^75^^,^ ^87^^–^ ^90^ and automation/ML (4 products, 2%).^91^^–^ ^94^ Finally, most of the methods guidance focused on producing evidence synthesis and evidence-based advice in routine circumstances (215 products, 87%) and only 19 (8%) addressed rapid approaches. Four of the included methods guidance were published in languages other than English (i.e., German, Spanish, and Portuguese).

**Figure 2:**
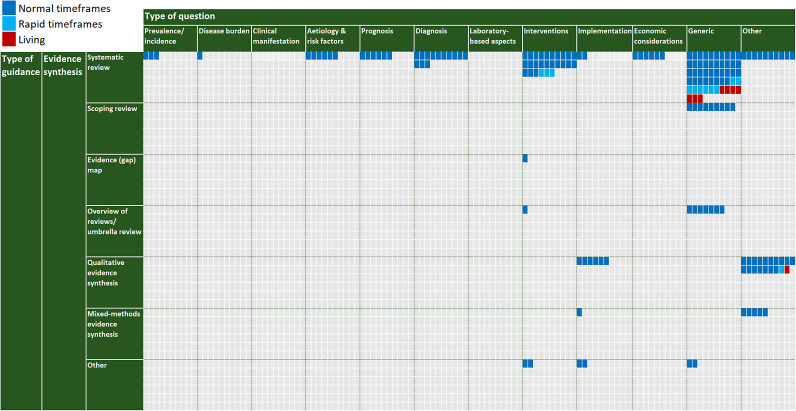
Map of evidence synthesis methods guidance. The interactive evidence map is available here: https://ew6.ibe.med.uni-muenchen.de/mediate/evidence-gap-map/. *Note*: Note: In this evidence map, the term “systematic review” is used to refer to systematic reviews of quantitative data (with or without meta-analysis), consistent with common usage. Qualitative and mixed-methods syntheses are presented as separate categories.

In the following, we describe the 247 methods guidance products included in the map. We describe the guidance products addressing evidence synthesis and evidence-based advice separately. For the guidance products on evidence synthesis, we further specify the type of question they address, as well as the specific product type and mode of production they describe (see Figure 2). This is further visually presented in an interactive map, which can be found at: https://ew6.ibe.med.uni-muenchen.de/mediate/evidence-gap-map/. In this map and data presentation, we use the term *systematic review*—as commonly understood—to refer to systematic reviews of quantitative data (with or without meta-analysis), distinguishing it from other forms of evidence synthesis such as qualitative evidence synthesis and mixed-methods syntheses. However, all of these represent systematic and structured approaches to synthesising evidence. For guidance products on evidence-based advice, we provide a summary of specific product type and production mode addressed; but do not specify the type of question as we omitted this specification for these guidance products.

### 3.4 Description of methods guidance on evidence synthesis

Our review identified significant variability in the availability and focus of methods guidance across different types of evidence synthesis questions. Below, we summarise the findings for each question type.

#### 3.4.1 Prevalence and incidence

We identified only three methods guidance products applicable to the questions of *prevalence/incidence* in evidence synthesis.^28^^,^ ^95^^,^ ^96^ Specifically, one offered methodological guidance for *systematic reviews* of observational epidemiological studies reporting prevalence and incidence data,^95^ the second described a broader framework (the PRECEPT framework) for evaluating and grading evidence in infectious disease epidemiology, prevention and control,^28^ and the third comprises the specific chapter on prevalence/incidence in the JBI Manual for Evidence Synthesis.^96^ All of these guidance products focused on conducting evidence synthesis in *normal* circumstances.

#### 3.4.2 Disease burden

The only identified methods guidance relevant to disease burden assessment was the PRECEPT framework.^28^

#### 3.4.3 Clinical manifestation

We did not identify any methods guidance specifically addressing evidence synthesis for clinical manifestation questions, such as symptomatology or disease progression.

#### 3.4.4 Aetiology and risk factors

We identified six methods guidance products applicable to the question of aetiology and risk factors in evidence synthesis.^46^^,^ ^96^^–^ ^100^ Examples include the COSMOS-E guidance^97^ and the specific chapter on aetiology/risk factors in the JBI Manual for Evidence Synthesis.^96^ All of these offered guidance on the conduct of *systematic reviews* of observational studies of aetiology in *normal* circumstances.

#### 3.4.5 Prognosis

Six methods guidance products addressed questions of prognosis.^25^^,^ ^101^^–^ ^105^ For example, the guidance by Riley et al. describes how various systematic review tools could be adapted for use across the steps of reviewing the prognostic factor studies.^103^ All of these offered guidance for conducting *systematic reviews* of prognostic studies in *normal* circumstances.

#### 3.4.6 Diagnosis

We found 13 methods guidance products applicable to the question of diagnosis.^28^^,^ ^101^^,^ ^104^^,^ ^106^^–^ ^115^ Examples include the Cochrane handbook for systematic reviews of diagnostic test accuracy (DTA)^109^ and the Template of Multiplicity and Analysis in Systematic Reviews proposed to be used in conjunction with the Cochrane DTA guidance.^114^ All of these focused on *systematic reviews* of diagnostic studies in *normal* circumstances.

#### 3.4.7 Laboratory-based aspects

We did not identify any methods guidance specifically addressing evidence synthesis for laboratory-based aspects, such as laboratory procedures.

#### 3.4.8 Interventions

We identified 30 methods guidance products applicable to interventions.^28^^,^ ^37^^,^ ^38^^,^ ^45^^,^ ^53^^,^ ^57^^,^ ^61^^,^ ^66^^,^ ^67^^,^ ^96^^,^ ^116^^–^ ^135^ Of these, 26 focused on *systematic reviews* of intervention effects,^28^^,^ ^37^^,^ ^38^^,^ ^45^^,^ ^53^^,^ ^57^^,^ ^61^^,^ ^66^^,^ ^96^^,^ ^116^^–^ ^123^^,^ ^125^^–^ ^133^ including the Cochrane,^38^ Campbell,^121^ and JBI guidance,^96^ one guidance from Campbell addressed *evidence (gap) maps*,^134^ one focused on *reviews of reviews* of interventions,^124^ and two were concerned with *other types of evidence synthesis.* ^67^^,^ ^135^ These included realist approaches, such as the RAMESES guidance for meta-narrative reviews.^135^ Most of these guidance products focused on *normal circumstances*, three addressed *rapid reviews.* ^61^^,^ ^122^^,^ ^130^ These included the original and updated guidance from the Cochrane Rapid Review Methods Group^122^^,^ ^130^ and one guidance focusing on rapid reviews for health policy and system questions.^61^

#### 3.4.9 Implementation

Ten methods guidance products were found applicable to implementation considera tions.^56^^,^ ^59^^,^ ^63^^,^ ^64^^,^ ^67^^,^ ^82^^,^ ^135^^–^ ^138^ Of these, two guidance products focused integrating implementation issues in *systematic reviews* ^59^^,^ ^82^ (here and throughout used to refer to reviews of quantitative data, unless otherwise specified), six guidance products from the Cochrane Qualitative and Implementation Methods Group addressed *qualitative evidence synthesis*,^56^^,^ ^63^^,^ ^64^^,^ ^136^^–^ ^138^ and one from the same group was concerned with *mixed-methods evidence synthesis.* ^56^ We also classified the two guidance products on realist approaches described under Interventions as applicable to implementation.^67^^,^ ^135^ All guidance products addressed evidence synthesis in *normal* circumstances.^56^^,^ ^59^^,^ ^63^^,^ ^64^^,^ ^67^^,^ ^82^^,^ ^135^^–^ ^138^

#### 3.4.10 Economic considerations

Six methods guidance products addressed economic considerations in evidence synthesis.^24^^,^ ^96^^,^ ^139^^–^ ^142^ Examples include the guidance on systematic reviews of economic evidence from the Community Preventive Services Task Force^143^ and the dedicated chapter from the JBI Manual for Evidence Synthesis.^96^ All focused on *systematic reviews* in *normal* circumstances.

#### 3.4.11 Generic

We classified 68 methods guidance products as generic, meaning they did not specify a type of question and/or covered many different questions.^26^^,^ ^30^^,^ ^31^^,^ ^36^^,^ ^43^^,^ ^48^^,^ ^60^^,^ ^62^^,^ ^79^^,^ ^92^^,^ ^94^^,^ ^143^^–^ ^198^ An example of such guidance is the methods guide on evidence synthesis for health policy and systems from WHO, highlighting various questions and synthesis methods.^60^ Of all the guidance products in this category, 52 were found to focus on *systematic reviews*,^26^^,^ ^30^^,^ ^31^^,^ ^36^^,^ ^43^^,^ ^48^^,^ ^60^^,^ ^62^^,^ ^92^^,^ ^94^^,^ ^143^^–^ ^148^^,^ ^150^^,^ ^152^^–^ ^154^^,^ ^156^^,^ ^157^^,^ ^159^^,^ ^160^^,^ ^162^^,^ ^163^^,^ ^165^^,^ ^167^^–^ ^169^^,^ ^172^^–^ ^178^^,^ ^180^^–^ ^182^^,^ ^184^^–^ ^186^^,^ ^188^^–^ ^191^^,^ ^194^^–^ ^198^ nine on *scoping reviews*,^79^^,^ ^149^^,^ ^155^^,^ ^161^^,^ ^166^^,^ ^170^^,^ ^171^^,^ ^192^^,^ ^193^ seven on *umbrella reviews*,^151^^,^ ^158^^,^ ^159^^,^ ^172^^,^ ^178^^,^ ^179^^,^ ^183^ and two on *other types of evidence synthesis.* ^164^^,^ ^187^ In the other category, one methods guidance product addressed realist meta-reviews,^164^ and one state-of-the-art literature review.^187^ Most methods guidance products in the generic category focused on evidence synthesis in *normal* circumstances, eight on *rapid* approaches,^30^^,^ ^31^^,^ ^94^^,^ ^156^^,^ ^167^^–^ ^169^^,^ ^185^ and seven on *living* approaches to evidence synthesis, including updating of evidence synthesis.^26^^,^ ^146^^,^ ^162^^,^ ^163^^,^ ^165^^,^ ^184^^,^ ^199^

#### 3.4.12 Other types of questions

We classified 29 methods guidance products as addressing *other types of questions* than those described above.^51^^,^ ^52^^,^ ^56^^,^ ^58^^,^ ^63^^,^ ^64^^,^ ^68^^,^ ^132^^,^ ^136^^–^ ^138^^,^ ^199^^–^ ^216^ Ten of these focused on *systematic reviews* and addressed issues, such as health state utility values, patient-reported outcome measures, toxicity factors, health communication, and human biomonitoring data.^58^^,^ ^132^^,^ ^200^^–^ ^205^^,^ ^210^^,^ ^212^ All of these focused on evidence synthesis in *normal* circumstances. Nineteen products focused on *qualitative evidence synthesis* ^51^^,^ ^56^^,^ ^58^^,^ ^63^^,^ ^64^^,^ ^136^^–^ ^138^^,^ ^199^^,^ ^206^^,^ ^207^^,^ ^209^^–^ ^216^ and five on *mixed-methods synthesis.* ^52^^,^ ^56^^,^ ^58^^,^ ^68^^,^ ^208^ Some of these were also relevant for implementation and therefore were also coded for that type of question (see above). We classified these as concerned with ‘other’ questions based on the suggestion from the MAG because most of these approaches address questions related to preferences and experiences and hence do not fit into any of the above categories. Most of these focused on synthesis in *normal circumstances*, one methods guidance was found on *rapid* qualitative evidence synthesis,^216^ and one on how to update qualitative synthesis (classified together with the guidance focusing on living approaches).^199^

### 3.5 Description of methods guidance on evidence-based advice

Of the 98 guidance products on evidence-based advice, 11 offered guidance on how to develop *HTAs*,^35^^,^ ^55^^,^ ^77^^,^ ^83^^–^ ^85^^,^ ^217^^–^ ^221^ such as the National Institute for Health and Care Excellence (NICE) manual for health technology evaluations.^221^ Only one of these, the HTA Core Model from the European Network for Health Technology Assessment (EUnetHTA), discussed a rapid development approach.^218^

Most of the guidance in this category (77 guidance products, 79%) focused on *guideline/recommendation* development for evidence-based practice,^23^^,^ ^27^^,^ ^29^^,^ ^31^^–^ ^34^^,^ ^39^^–^ ^42^^,^ ^44^^,^ ^49^^,^ ^54^^,^ ^70^^–^ ^76^^,^ ^78^^,^ ^80^^,^ ^81^^,^ ^86^^–^ ^91^^,^ ^93^^,^ ^133^^,^ ^154^^,^ ^167^^,^ ^176^^,^ ^207^^,^ ^222^^–^ ^262^ such as the GRADE guidance^133^ and the ADAPTE methodology,^87^ and 10 specifically focused on *EtD considerations*,^3^^,^ ^34^^,^ ^40^^,^ ^47^^,^ ^71^^,^ ^73^^,^ ^243^^,^ ^263^^–^ ^265^ including the GRADE EtD^47^ and the WHO-INTEGRATE frameworks.^3^ Most of these included products from key institutions and entities, such as the GRADE Working Group, WHO, NICE, the Guidelines International Network (GIN), and the US Prevention Services Task Force. Only eight of these guidance products addressed *rapid* approaches to guideline/recommendation development,^27^^,^ ^29^^,^ ^31^^,^ ^88^^,^ ^167^^,^ ^223^^,^ ^245^^,^ ^260^ such as the extension of the GIN-McMaster guideline development checklist for rapid recommendations,^245^ and seven offered guidance on *living guidelines*,^90^^,^ ^93^^,^ ^222^^,^ ^236^^,^ ^238^^,^ ^239^^,^ ^249^ including the guidance produced by the Australian Living Evidence Consortium in partnership with NICE and the US GRADE network.^93^^,^ ^236^^,^ ^239^^,^ ^249^ Six further products offered guidance on *other* types of evidence-based advice,^225^^,^ ^227^^,^ ^228^^,^ ^244^^,^ ^266^^,^ ^267^ including how to develop briefings to support evidence-informed decision-making and actionable statements.

## 4 Discussion

### 4.1 Summary of the main findings

This scoping review provides a comprehensive overview of existing methods guidance for evidence synthesis and evidence-based advice employed in public health, as well as in clinical medicine and health care. Through a systematic and comprehensive search and consultations with methodologists and public health professionals, the map identified 391 guidance documents. Among these, 247 were classified as guidance products, while the remainder were categorised as guidance-like products. Most of the guidance products addressed evidence synthesis (155 products), with relatively fewer focusing on evidence-based advice (98 products). Additionally, the identified methods guidance often lacked specificity for public health contexts and infectious diseases. While we identified many areas lacking guidance, this does not necessarily indicate a need to produce new guidance. In some areas it might be reasonable to have limited guidance. For example, knowledge regarding the aetiology of well-known diseases does not need to be constantly updated, and one would therefore not expect any methods guidance for conducting living reviews on aetiology questions. Below we summarise and discuss the gaps that we think might benefit from further method development.

#### 4.1.1 Evidence synthesis

The map demonstrates that the most comprehensive methods guidance exists for evidence synthesis related to interventions (30 products) and diagnostics (13 products). Some guidance is also available for prognosis (6 products) and aetiology/risk factors (6 products). However, gaps were identified in areas such as clinical manifestation and laboratory-based aspects, for which no guidance was found. Guidance for prevalence/incidence and disease burden is rare, suggesting a potential area for further guidance development to support a broader range of public health questions.

Additionally, most methods guidance focuses on systematic reviews of quantitative data, with limited attention to other types of evidence synthesis, such as qualitative evidence synthesis, scoping reviews, umbrella reviews, and mixed-methods synthesis. Expanding the scope of guidance to encompass these methodologies may be useful to address the diverse and complex needs of public health decision-making.

Finally, while most guidance products are designed for routine circumstances, there is limited attention to urgent or resource-constrained contexts. Although some guidance for rapid synthesis exists, there is a notable gap in ultra-rapid synthesis approaches (e.g., within 48 hours), which may better align with the pace required by many public health institutions during emergency responses.^268^ This need has been explicitly raised by ECDC in the context of commissioning this work, based on internal communications and expressed institutional priorities. This highlights the importance of developing further resources to support evidence synthesis in emergency scenarios.

#### 4.1.2 Evidence-based advice

For evidence-based advice, most guidance products focus on the development of guidelines, evidence-based recommendations, and evidence-to-decision frameworks. There is relatively limited guidance on other types of evidence-based advice, such as HTAs. There may thus be an opportunity to broaden the range of guidance products available for decision-makers.

Similar to evidence synthesis, most guidance for evidence-based advice is intended for routine, non-urgent circumstances. While some resources are emerging for rapid guideline development and living guidelines, there is a complete lack of ultra-rapid approaches. Diversifying methods guidance to include these formats may help address the dynamic and time-sensitive nature of public health emergencies.

### 4.2 Strengths and limitations of the map

By systematically charting existing guidance, this interactive map serves as the first and most up-to-date repository of methods guidance on evidence synthesis and evidence-based advice for methodologists and decision-makers in public health, as well as clinical medicine and health care in general. It also provides a solid foundation for identifying priorities and supporting further methodological development by highlighting critical gaps. Despite the comprehensive approach undertaken in developing this map, several limitations may impact the completeness of the findings. The inclusion criteria for methods guidance were intentionally broad to capture a wide range of documents across different types of evidence synthesis and evidence-based advice. However, by focusing on guidance that addresses the overall process rather than specific steps (e.g., critical appraisal of studies, approaches to evidence synthesis), more innovative methodological developments related to individual steps, including those making use of artificial intelligence, might have been excluded. Our searches were conducted in April 2024, and it is possible that more recent tools and frameworks were not yet widely disseminated or indexed at the time. This could result in underrepresentation of methodological nuances that are critical for certain types of evidence synthesis or advice. However, focusing on guidance regarding the overall process was necessary to maintain this scoping review feasible.

Furthermore, the searches were conducted in English, focusing on scientific databases and the websites of key institutions, which may have led to the exclusion of relevant guidance published in other languages or formats less likely to be indexed or hosted on institutional platforms (e.g., textbooks). However, all identified non-English documents were examined and included in the map when relevant. The development of the map involved subjective decisions during the mapping and categorisation of the guidance documents. Although these decisions were made using a predefined framework, validated through pilot testing, discussed within the research team where necessary and performed by an experienced team of methodologists, there is an inherent risk of misclassification or oversimplification, especially given the diverse nature of the documents included. We also did not involve any stakeholders in developing the coding framework for the map. Moreover, given the large volume of methods guidance identified, certain planned aspects of the mapping process, such as detailed data extraction on reported experiences and limitations, were not feasible.

Finally, we did not initially plan for this map to be a living resource. However, its straightforward design allows for efficient updates. The team is currently exploring mechanisms and collaborations to transition it into a living format to ensure its ongoing relevance and utility.

### 4.3 Insights for further methods development

This scoping review was undertaken to systematically identify and map existing methods guidance for evidence synthesis and evidence-based advice, with the overarching aim of informing areas for methods development in public health. The review findings align with the concerns raised by many public health institutions, highlighting the need for methods guidance that recognises the complexities of synthesising evidence from public health policy and systems research.^60^ The need has also been underscored for guidance that supports decision-making under uncertainties and within constrained timeframes, as is often required in public health emergencies.^1^^,^ ^269^ Our findings reaffirm these by showing that existing guidance is predominantly designed for clinical medicine and routine decision-making contexts, rather than the dynamic and multifaceted landscape of public health.

The findings present a solid basis for further steps. One focus could be exploring how systematic and transparent approaches for evidence synthesis and evidence-based advice development can be effectively adapted to rapid and ultra-rapid timeframes. While methods guidance for rapid evidence synthesis on studies of interventions has been recently published,^130^ a significant challenge remains in ensuring methodological rigour while reducing the timeframe, particularly for ultra-rapid decision-making (i.e., within 48 hours in emergencies) and for rapidly synthesising other types of primary studies (e.g., diagnostic test accuracy, complex public health interventions, qualitative evidence). Future efforts within ECDC could examine pragmatic approaches, such as streamlined data screening and extraction, as well as integration of structured expert judgement in time-sensitive situations. Collaborative models for evidence-informed practice that enhance efficiency and reduce redundancies are currently being supported by institutions, such as the National Collaborating Centre for Public Health in Canada^270^ and the Global Evidence Commission at the international level.^271^ Exploring similar models in the European context could help strengthen the methodological infrastructure for evidence-based public health decision-making in the region.

More broadly, the field of evidence-based public health would benefit from expanding synthesis methodologies beyond traditional intervention (effectiveness) studies. While many guidance products in our map were classified as addressing generic questions, we observed during the screening and coding process that much of this guidance implicitly reflects assumptions and frameworks derived from the intervention-effectiveness paradigm—for example, through the use of the PICO format for formulating questions based on population, intervention, comparison and outcomes, and emphasis on randomised controlled trials. Public health decisions frequently depend on diverse types of evidence, including data on disease burden, implementation feasibility, contextual acceptability, and real-world adaptability.^3^ Future methodological work could explore how these multiple streams of evidence can be efficiently synthesised in a time-sensitive manner. Additionally, emerging technologies such as artificial intelligence-assisted synthesis offer promising avenues for accelerating evidence synthesis while also maintaining transparency and rigour.^272^ A dialogue among methodologists, public health institutions, practitioners, and policymakers will be essential to refining approaches that are both robust and responsive to the complexities of real-world public health decision-making.

## Supporting information

Movsisyan et al. supplementary material Movsisyan et al. supplementary material

## Data Availability

All data contributing to this manuscript are derived from published sources identified through systematic searches of scientific databases and institutional websites. The full list of included references and corresponding extracted data can be explored via the interactive evidence map, accessible at https://ew6.ibe.med.uni-muenchen.de/mediate/evidence-gap-map/. References for included publications are also provided in the manuscript.

## Supplementary material

To view supplementary material for this article, please visit http://doi.org/10.1017/rsm.2025.10051.
