# Supplementary material for: Conducting evidence synthesis and developing evidence-based advice in public health and beyond: A scoping review and map of methods guidance
Source: Res Synth Methods. 2025 Nov 18;17(2):240–64. doi: 10.1017/rsm.2025.10051 (PMC12873621; doi:10.1017/rsm.2025.10051)
Supplement: Movsisyan et al. supplementary material [file S1759287925100513sup001.docx]

# File S1: 26 key institutions

1. Africa CDC
2. Australian Commission on Safety and Quality in Health Care
3. Canadian Task Force on Preventive Health Care
4. Campbell Collaboration
5. Cochrane Collaboration
6. EQUATOR Network
7. Guidelines International Network (GIN)
8. GRADE Working Group
9. Joanna Briggs Institute
10. National Health and Medical Research Council (NHMRC)
11. Public Health Agency of Canada
12. Healthcare Improvement Scotland: Scottish Intercollegiate Guidelines Network (SIGN)
13. Social Care Institute for Excellence (SCIE)
14. UK Health Security Agency
15. UK NICE
16. United States Agency for Healthcare Research and Quality
17. US CDC
18. United States Community Preventive Services Task Force
19. World Health Organization
20. Independent Institute for Quality and Efficiency in Health Care (Institut für Qualität und Wirtschaftlichkeit im Gesundheitswesen, IQWiG )
21. European Network for Health Technology Assessment
22. International Network of Agencies for Health Technology Assessment ((EUnetHTA)
23. Norwegian Institute of Public Health
24. Public Health Agency of Sweden
25. National Institute for Public Health and the Environment (RIVM) – Netherlands
26. Appraisal of Guidelines for Research and Evaluation (AGREE) Trust

# File S2: Review search strategy

The Medline search strategy was assessed by a second information specialist using the Peer Review of Electronic Search Strategies (PRESS) guideline.

| **Concept** | **#** | **Searches** | **Results** |
| --- | --- | --- | --- |
| A. evidence synthesis as topic | 1 | *"review literature as topic"/ or *systematic reviews as topic/ | 5485 |
|  | 2 | (systematic* adj3 (reviews or reviewing)).ti,bt,kf. | 7980 |
|  | 3 | (Systematic overviews or "reviews of reviews" or "overviews of reviews" or quantitative syntheses or research syntheses or Systematic literature research or meta-analyses or metaanalyses or meta-analytic* reviews or meta-reviews or meta-syntheses or metasyntheses or meta-ethnographies or Health technology assessments or HTAs).ti,bt,kf. | 6430 |
|  | 4 | ((Cochrane or systemic or scoping or mapping or umbrella or effectiveness or quantitative) adj reviews).ti,bt,kf. | 642 |
|  | 5 | (evidence adj2 (maps or syntheses)).ti,bt,kf. | 126 |
|  | 6 | ((mixed-method? or qualitativ* or integrat* or pragmatic or rapid) adj2 (reviews or overviews or syntheses)).ti,bt,kf. | 283 |
|  | 7 | (Rapid adj6 (appraisals or assessments or syntheses) adj6 (evidence or literature)).ti,bt,kf. | 4 |
|  | 8 | ((systematic or Cochrane or scoping or mapping or effectiveness or quantitative or evidence or mixed-method? or qualitativ* or integrat* or pragmatic or rapid) adj2 (reviews or syntheses or reviewing)).ab. /freq=3 | 5830 |
|  | 9 | ("reviews of reviews" or "overviews of reviews" or "umbrella reviews").ab. /freq=3 | 28 |
|  | 10 | (meta-analyses or metaanalyses or meta-analytic* reviews or meta-reviews or meta-syntheses or metasyntheses or meta-ethnographies or Health technology assessments or HTAs or research syntheses).ab. /freq=3 | 3520 |
|  | 11 | evidence based public health.ti,bt,ab,kf. | 569 |
|  | 12 | or/1-11 | 21951 |
| B. methodology | 13 | Research Report/st [Standards] | 1183 |
|  | 14 | Evidence-Based Medicine/mt [Methods] | 5880 |
|  | 15 | Research Design/ | 127830 |
|  | 16 | Epidemiologic Methods/ | 31618 |
|  | 17 | Research/ | 205105 |
|  | 18 | (method* or conduct* or develop* or undertak* or perform* or prepar* or produc* or creat* or design* or reporting).ti,bt. | 2515405 |
|  | 19 | (conduct* or develop* or undertak* or perform* or prepar* or produc* or creat* or design* or reporting).ab. /freq=3 | 3399145 |
|  | 20 | method*.ab. /freq=3 | 697620 |
|  | 21 | ((review* or synthes?s or meta-analys?s or metaanalys?s or meta-analytic* review* or meta-review? or meta-synthes?s or metasynthes?s or meta-ethnograph* or Health technology assessment?) adj3 method*).kf. | 557 |
|  | 22 | method*.jw. | 197737 |
|  | 23 | or/13-22 | 5823341 |
| A+B | 24 | 12 and 23 | 10923 |
| C. Guidance | 25 | Manuals as Topic/ | 3714 |
|  | 26 | Guideline.pt. | 16385 |
|  | 27 | Checklist/ | 9109 |
|  | 28 | (manual? or guidance or guideline? or guide? or primer? or "how to" or tutorial? or recommendation? or handbook? or framework? or checklist? or hand-book? or frame-work? or check-list? or step? or white paper? or toolkit? or tool-kit?).ti,bt,kf. | 675063 |
|  | 29 | ((best or code or good) adj2 (practice or practices)).ti,bt,kf. | 12052 |
|  | 30 | (manual or guidance or guideline or guide or primer or tutorial or handbook or hand-book).ab. /freq=3 | 49865 |
|  | 31 | ((best or code or good) adj2 (practice or practices)).ab. /freq=3 | 2248 |
|  | 32 | or/25-31 | 722768 |
| A+B+C | 33 | 24 and 32 | 1715 |
| D. Guidance for producing evidence synteses | 34 | ((systematic or Cochrane or scoping or mapping or umbrella or effectiveness or overview or integrative or pragmatic or rapid) adj3 (reviews or syntheses or reviewing) adj6 (method* or conduct* or develop* or undertak* or perform* or prepar* or produc* or creat* or design* or report* or manual? or guidance or guideline? or guide? or primer? or "how to" or tutorial? or recommendation? or handbook? or framework? or checklist? or hand-book? or frame-work? or check-list? or step? or white paper? or toolkit? or tool-kit?)).kf. | 446 |
|  | 35 | ((meta-analyses or metaanalyses or meta-analytic* reviews or meta-reviews or meta-syntheses or metasyntheses or meta-ethnograph* or Health technology assessments) adj6 (method* or conduct* or develop* or undertak* or perform* or prepar* or produc* or creat* or design* or report* or manual? or guidance or guideline? or guide? or primer? or "how to" or tutorial? or recommendation? or handbook? or framework? or checklist? or hand-book? or frame-work? or check-list? or step? or white paper? or toolkit? or tool-kit?)).kf. | 225 |
|  | 36 | (("conducting a?" or "developing a?" or "undertaking a?" or "performing a?" or "producing a?" or "preparing a?" or "designing a?") adj3 (review or meta-analysis or metaanalysis or synthesis or overview or evidence)).ti,bt,kf. | 279 |
|  | 37 | ((((systematic or Cochrane or scoping or mapping or umbrella or qualitativ* or integrat* or pragmatic or rapid or meta-analytic) adj (review or synthesis)) or meta-analysis or metaanalysis or meta-review or meta-synthesis or metasynthesis or meta-ethnography or Health technology assessment or HTA) adj (methods or methodology or development or process or manual or guidance or guideline or guide or primer or tutorial or handbook or framework or hand-book? or frame-work? or toolkit or tool-kit)).ti,bt. | 239 |
|  | 38 | ((systematic or Cochrane or scoping or mapping or umbrella or qualitativ* or integrat* or pragmatic or rapid or meta-analytic or evidence) adj2 (review* or synthes?s) adj6 (methods or methodology or development or process or design or manual or guidance or guideline or guide or primer or tutorial or handbook or framework or hand-book? or frame-work? or toolkit or tool-kit or introduction)).ti,bt. and (((systematic or Cochrane or scoping or mapping or umbrella or qualitativ* or integrat* or pragmatic or rapid or meta-analytic) adj (review* or synthes?s)) or SR or SRs or RR or RRs).ab. /freq=3 | 899 |
|  | 39 | ((meta-analysi? or metaanalys?s or meta-review? or meta-synthes?s or metasynthes?s or meta-ethnograph* or Health technology assessment? or HTA?) adj6 (methods or methodology or development or process or design or manual or guidance or guideline or guide or primer or tutorial or handbook or framework or hand-book? or frame-work? or toolkit or tool-kit or introduction)).ti,bt. and (meta-analysi? or metaanalys?s or meta-review? or meta-synthes?s or metasynthes?s or meta-ethnograph* or Health technology assessment? or HTA? or MA or MAs).ab. /freq=3 | 444 |
| Part 1: (A+B+C) or D | 40 | or/33-39 | 3581 |
| E. guideline development | 41 | ((conduct* or develop* or undertak* or perform* or prepar* or produc* or creat* or design* or reporting) adj6 (guideline? or guidance or research agenda? or Global strateg* or Regional strateg*)).ti,bt,kf. and (public health or evidence).mp. | 2484 |
|  | 42 | ((conduct* or develop* or undertak* or perform* or prepar* or produc* or creat* or design* or reporting) adj6 (Horizon scan? or policy brief? or policy formulation?)).ti,bt,kf. | 14 |
|  | 43 | ((conduct* or develop* or undertak* or perform* or prepar* or produc* or creat* or design* or reporting) adj6 (recommend* or policy or policies or decision aid? or decision mak* or decisionmak* or decision support*)).ti,bt,kf. and (public health or evidence).mp. | 2684 |
|  | 44 | (public health adj3 (guidelines or recommendations or guidance or Horizon scans or policy briefs or policy formulations)).ti,bt,kf. | 482 |
|  | 45 | or/41-44 | 5515 |
| F. methodology | 46 | exp Guidelines as Topic/st [Standards] | 12216 |
|  | 47 | Manuals as Topic/ | 3714 |
|  | 48 | Evidence-Based Medicine/ | 76367 |
|  | 49 | (method* or manual? or primer? or "how to" or tutorial? or handbook? or framework? or checklist? or hand-book? or frame-work? or check-list? or step? or white paper? or toolkit? or tool-kit?).ti,bt,kf. | 1038706 |
|  | 50 | ((best or code or good) adj2 (practice or practices)).ti,bt,kf. | 12052 |
|  | 51 | method*.ab. /freq=3 | 697620 |
|  | 52 | (method* or policy or policies or systems or health services).jw. | 320060 |
|  | 53 | or/46-52 | 1912793 |
| E+F | 54 | 45 and 53 | 2199 |
| G. Guideline development Manuals | 55 | ((guideline? or guidance) adj develop* adj3 (method* or manual? or primer? or "how to" or handbook? or framework? or checklist? or hand-book? or frame-work? or check-list? or step? or tutorial? or white paper? or toolkit? or tool-kit?)).ti,bt,kf. | 64 |
|  | 56 | ((guideline? or guidance) adj develop* adj3 (method* or manual? or primer? or "how to" or handbook? or framework? or checklist? or hand-book? or frame-work? or check-list? or step? or tutorial? or white paper? or toolkit? or tool-kit?)).ab. /freq=2 | 61 |
|  | 57 | ((guideline? or guidance or recommendations) adj2 (manual or handbook or hand-book)).ti,bt,kf. | 118 |
|  | 58 | ((evidence based or evidence informed or clinical or practice or public health or regulatory or emergency response or ethic* or implementation) adj (guideline? or guidance or recommendation?)).ti,bt,kf. | 25288 |
|  | 59 | ((evidence based or evidence informed or clinical or practice or public health) adj3 (Horizon scan? or policy brief? or policy formulation? or research agenda? or Global strateg* or Regional strateg*)).ti,bt,kf. | 141 |
|  | 60 | (method* or conduct* or develop* or undertak* or perform* or prepar* or produc* or creat* or design* or reporting).ti,bt,kf. | 2815289 |
|  | 61 | 53 and (58 or 59) and 60 | 1643 |
| Part 2: (E+F) or G | 62 | or/54-57,61 | 3342 |
| (A+B+C) or D or (E+F) or G | 63 | 40 or 62 | 6694 |
| **Total, limited by date** | 64 | limit 63 to yr="2000 -Current" | 6439 |

## Web of Science Core Collection (Science Citation Index Expanded (SCI-EXPANDED), Social Sciences Citation Index (SSCI), Arts & Humanities Citation Index (AHCI), Emerging Sources Citation Index (ESCI))

24.04.2024

| **Concept** | **#** | **Search Query** | **Results** |
| --- | --- | --- | --- |
| A. evidence synthesis as topic | 1 | TI=(systematic* NEAR/3 ("reviews" OR "reviewing")) OR AK=(systematic* NEAR/3 ("reviews" OR "reviewing")) OR KP=(systematic* NEAR/3 ("reviews" OR "reviewing")) | 13492 |
|  | 2 | TI=("Systematic overviews" OR "reviews of reviews" OR "overviews of reviews" OR "quantitative syntheses" OR "research syntheses" OR "Systematic literature research" OR "meta-analyses" OR "metaanalyses" OR "meta-analytic* reviews" OR "meta-reviews" OR "meta-syntheses" OR "metasyntheses" OR "meta-ethnographies" OR "Health technology assessments" OR "HTAs") OR AK=("Systematic overviews" OR "reviews of reviews" OR "overviews of reviews" OR "quantitative syntheses" OR "research syntheses" OR "Systematic literature research" OR "meta-analyses" OR "metaanalyses" OR "meta-analytic* reviews" OR "meta-reviews" OR "meta-syntheses" OR "metasyntheses" OR "meta-ethnographies" OR "Health technology assessments" OR "HTAs") OR KP=("Systematic overviews" OR "reviews of reviews" OR "overviews of reviews" OR "quantitative syntheses" OR "research syntheses" OR "Systematic literature research" OR "meta-analyses" OR "metaanalyses" OR "meta-analytic* reviews" OR "meta-reviews" OR "meta-syntheses" OR "metasyntheses" OR "meta-ethnographies" OR "Health technology assessments" OR "HTAs") | 9974 |
|  | 3 | TI=((Cochrane OR systemic OR scoping OR mapping OR umbrella OR effectiveness OR quantitative) NEAR/0 "reviews") OR AK=((Cochrane OR systemic OR scoping OR mapping OR umbrella OR effectiveness OR quantitative) NEAR/0 "reviews") OR KP=((Cochrane OR systemic OR scoping OR mapping OR umbrella OR effectiveness OR quantitative) NEAR/0 "reviews") | 803 |
|  | 4 | TI=(evidence NEAR/2 ("maps" OR "syntheses")) OR AK=(evidence NEAR/2 ("maps" OR "syntheses")) OR KP=(evidence NEAR/2 ("maps" OR "syntheses")) | 206 |
|  | 5 | TI=((mixed-method$ OR qualitativ* OR integrat* OR pragmatic OR rapid) NEAR/2 ("reviews" OR "overviews" OR "syntheses")) OR AK=((mixed-method$ OR qualitativ* OR integrat* OR pragmatic OR rapid) NEAR/2 ("reviews" OR "overviews" OR "syntheses")) OR KP=((mixed-method$ OR qualitativ* OR integrat* OR pragmatic OR rapid) NEAR/2 ("reviews" OR "overviews" OR "syntheses")) | 513 |
|  | 6 | TI=(Rapid NEAR/6 ("appraisals" OR "assessments" OR "syntheses") NEAR/6 (evidence OR literature)) OR AK=(Rapid NEAR/6 ("appraisals" OR "assessments" OR "syntheses") NEAR/6 (evidence OR literature)) OR KP=(Rapid NEAR/6 ("appraisals" OR "assessments" OR "syntheses") NEAR/6 (evidence OR literature)) | 8 |
|  | 7 | TS=("evidence based public health") | 538 |
|  | 8 | #7 OR #6 OR #5 OR #4 OR #3 OR #2 OR #1 | 22865 |
| B. methodology | 9 | TI=(method* OR conduct* OR develop* OR undertak* OR perform* OR prepar* OR produc* OR creat* OR design* OR reporting) OR AK=((review* OR synthes$s OR meta-analys$s OR metaanalys$s OR "meta-analytic* review*" OR meta-review$ OR meta-synthes$s OR metasynthes$s OR meta-ethnograph* OR "Health technology assessment$") NEAR/3 method*) OR KP=((review* OR synthes$s OR meta-analys$s OR metaanalys$s OR "meta-analytic* review*" OR meta-review$ OR meta-synthes$s OR metasynthes$s OR meta-ethnograph* OR "Health technology assessment$") NEAR/3 method*) OR SO=(method*) | 7391132 |
| A+B | 10 | #9 AND #8 | 3823 |
| C. Guidance | 11 | TI=(manual$ OR guidance OR guideline$ OR guide$ OR primer$ OR "how to" OR tutorial$ OR recommendation$ OR handbook$ OR framework$ OR checklist$ OR hand-book$ OR frame-work$ OR check-list$ OR step$ OR "white paper$" OR toolkit$ OR tool-kit$) OR AK=(manual$ OR guidance OR guideline$ OR guide$ OR primer$ OR "how to" OR tutorial$ OR recommendation$ OR handbook$ OR framework$ OR checklist$ OR hand-book$ OR frame-work$ OR check-list$ OR step$ OR "white paper$" OR toolkit$ OR tool-kit$) OR KP=(manual$ OR guidance OR guideline$ OR guide$ OR primer$ OR "how to" OR tutorial$ OR recommendation$ OR handbook$ OR framework$ OR checklist$ OR hand-book$ OR frame-work$ OR check-list$ OR step$ OR "white paper$" OR toolkit$ OR tool-kit$) | 1744304 |
|  | 12 | TI=((best OR code OR good) NEAR/2 (practice OR practices)) OR AK=((best OR code OR good) NEAR/2 (practice OR practices)) OR KP=((best OR code OR good) NEAR/2 (practice OR practices)) | 23947 |
|  | 13 | #12 OR #11 | 1763523 |
| A+B+C | 14 | #13 AND #10 | 1002 |
| D. Guidance for producing evidence synteses | 15 | AK=(((systematic OR Cochrane OR scoping OR mapping OR umbrella OR effectiveness OR overview OR integrative OR pragmatic OR rapid) NEAR/3 (reviews OR syntheses OR reviewing) NEAR/6 (method* OR conduct* OR develop* OR undertak* OR perform* OR prepar* OR produc* OR creat* OR design* OR report* OR manual$ OR guidance OR guideline$ OR guide$ OR primer$ OR "how to" OR tutorial$ OR recommendation$ OR handbook$ OR framework$ OR checklist$ OR hand-book$ OR frame-work$ OR check-list$ OR step$ OR "white paper$" OR toolkit$ OR tool-kit$))) | 236 |
|  | 16 | AK=(((meta-analyses OR metaanalyses OR "meta-analytic* reviews" OR meta-reviews OR meta-syntheses OR metasyntheses OR meta-ethnograph* OR "Health technology assessments") NEAR/6 (method* OR conduct* OR develop* OR undertak* OR perform* OR prepar* OR produc* OR creat* OR design* OR report* OR manual$ OR guidance OR guideline$ OR guide$ OR primer$ OR "how to" OR tutorial$ OR recommendation$ OR handbook$ OR framework$ OR checklist$ OR hand-book$ OR frame-work$ OR check-list$ OR step$ OR "white paper$" OR toolkit$ OR tool-kit$))) | 100 |
|  | 17 | ((TI=((("conducting a$" OR "developing a$" OR "undertaking a$" OR "performing a$" OR "producing a$" OR "preparing a$" OR "designing a$") NEAR/3 (review OR meta-analysis OR metaanalysis OR synthesis OR overview OR evidence)))) OR AK=((("conducting a$" OR "developing a$" OR "undertaking a$" OR "performing a$" OR "producing a$" OR "preparing a$" OR "designing a$") NEAR/3 (review OR meta-analysis OR metaanalysis OR synthesis OR overview OR evidence)))) OR KP=((("conducting a$" OR "developing a$" OR "undertaking a$" OR "performing a$" OR "producing a$" OR "preparing a$" OR "designing a$") NEAR/3 (review OR meta-analysis OR metaanalysis OR synthesis OR overview OR evidence))) | 444 |
|  | 18 | TI=(((((systematic OR Cochrane OR scoping OR mapping OR umbrella OR qualitativ* OR integrat* OR pragmatic OR rapid OR meta-analytic) NEAR/0 (review OR synthesis)) OR meta-analysis OR metaanalysis OR meta-review OR meta-synthesis OR metasynthesis OR meta-ethnography OR "Health technology assessment" OR HTA) NEAR/0 (methods OR methodology OR development OR process OR manual OR guidance OR guideline OR guide OR primer OR tutorial OR handbook OR framework OR hand-book$ OR frame-work$ OR toolkit OR tool-kit))) | 1492 |
| E. Topic: Guidance for evidence synteses | 19 | TS=((manual$ OR guidance OR guideline$ OR guide$ OR primer$ OR "how to" OR tutorial$ OR recommendation$ OR handbook$ OR framework$ OR checklist$ OR hand-book$ OR frame-work$ OR check-list$ OR step$ OR "white paper$" OR toolkit$ OR tool-kit$) NEAR/2 (method* OR conduct* OR develop* OR undertak* OR perform* OR prepar* OR produc* OR creat* OR design* OR report*) NEAR/2 (systematic OR Cochrane OR scoping OR mapping OR umbrella OR effectiveness OR overview OR integrative OR pragmatic OR rapid) NEAR/2 (review$ OR synthes$s OR reviewing) ) | 3896 |
|  | 20 | TS=((manual$ OR guidance OR guideline$ OR guide$ OR primer$ OR "how to" OR tutorial$ OR recommendation$ OR handbook$ OR framework$ OR checklist$ OR hand-book$ OR frame-work$ OR check-list$ OR step$ OR "white paper$" OR toolkit$ OR tool-kit$) NEAR/2 (method* OR conduct* OR develop* OR undertak* OR perform* OR prepar* OR produc* OR creat* OR design* OR report*) NEAR/2 (meta-analy* OR metaanaly* OR meta-review$ OR meta-synthes$s OR metasynthes$s OR meta-ethnograph* OR "Health technology assessment*") ) | 2179 |
|  | 21 | #20 OR #19 | 5860 |
| F. Public health | 22 | WC=(Public, Environmental & Occupational Health OR Health Care Sciences & Services OR Health Policy & Services) OR (TS=("public health" OR "health care" OR healthcare OR (health* NEAR/2 emergenc*) OR "health systems" OR "health polic*")) OR SO=("public health" OR "health systems" OR "health polic*") | 2619602 |
| E+F | 23 | #22 AND #21 | 1874 |
| Part 1: (A+B+C) or D or (E+F) | 24 | #23 OR #18 OR #17 OR #16 OR #15 OR #14 | 4864 |
| G. guideline development | 25 | ((TI=(((conduct* OR develop* OR undertak* OR perform* OR prepar* OR produc* OR creat* OR design* OR reporting) NEAR/6 (guideline$ OR guidance OR "research agenda$" OR "Global strateg*" OR "Regional strateg*")) )) OR AK=(((conduct* OR develop* OR undertak* OR perform* OR prepar* OR produc* OR creat* OR design* OR reporting) NEAR/6 (guideline$ OR guidance OR "research agenda$" OR "Global strateg*" OR "Regional strateg*")) )) OR KP=(((conduct* OR develop* OR undertak* OR perform* OR prepar* OR produc* OR creat* OR design* OR reporting) NEAR/6 (guideline$ OR guidance OR "research agenda$" OR "Global strateg*" OR "Regional strateg*")) ) | 15074 |
|  | 26 | ((TI=(((conduct* OR develop* OR undertak* OR perform* OR prepar* OR produc* OR creat* OR design* OR reporting) NEAR/6 ("Horizon scan$" OR "policy brief$" OR "policy formulation$")))) OR AK=(((conduct* OR develop* OR undertak* OR perform* OR prepar* OR produc* OR creat* OR design* OR reporting) NEAR/6 ("Horizon scan$" OR "policy brief$" OR "policy formulation$")))) OR KP=(((conduct* OR develop* OR undertak* OR perform* OR prepar* OR produc* OR creat* OR design* OR reporting) NEAR/6 ("Horizon scan$" OR "policy brief$" OR "policy formulation$"))) | 48 |
|  | 27 | ((TI=((conduct* OR develop* OR undertak* OR perform* OR prepar* OR produc* OR creat* OR design* OR reporting) NEAR/6 (recommend* OR policy OR policies OR "decision aid$" OR "decision mak*" OR decisionmak* OR "decision support*"))) OR AK=((conduct* OR develop* OR undertak* OR perform* OR prepar* OR produc* OR creat* OR design* OR reporting) NEAR/6 (recommend* OR policy OR policies OR "decision aid$" OR "decision mak*" OR decisionmak* OR "decision support*"))) OR KP=((conduct* OR develop* OR undertak* OR perform* OR prepar* OR produc* OR creat* OR design* OR reporting) NEAR/6 (recommend* OR policy OR policies OR "decision aid$" OR "decision mak*" OR decisionmak* OR "decision support*")) | 40579 |
|  | 28 | TI=("public health" NEAR/3 (guidelines OR recommendations OR guidance OR "Horizon scans" OR "policy briefs" OR "policy formulations")) OR AK=("public health" NEAR/3 (guidelines OR recommendations OR guidance OR "Horizon scans" OR "policy briefs" OR "policy formulations")) OR KP=("public health" NEAR/3 (guidelines OR recommendations OR guidance OR "Horizon scans" OR "policy briefs" OR "policy formulations")) | 626 |
|  | 29 | #28 OR #27 OR #26 OR #25 | 55743 |
| H. methodology | 30 | TI=(method* OR manual$ OR primer$ OR "how to" OR tutorial$ OR handbook$ OR framework$ OR checklist$ OR hand-book$ OR frame-work$ OR check-list$ OR step$ OR "white paper$" OR toolkit$ OR tool-kit$) OR AK=(method* OR manual$ OR primer$ OR "how to" OR tutorial$ OR handbook$ OR framework$ OR checklist$ OR hand-book$ OR frame-work$ OR check-list$ OR step$ OR "white paper$" OR toolkit$ OR tool-kit$) OR KP=(method* OR manual$ OR primer$ OR "how to" OR tutorial$ OR handbook$ OR framework$ OR checklist$ OR hand-book$ OR frame-work$ OR check-list$ OR step$ OR "white paper$" OR toolkit$ OR tool-kit$) | 3245076 |
|  | 31 | TI=((best OR code OR good) NEAR/2 (practice OR practices)) OR AK=((best OR code OR good) NEAR/2 (practice OR practices)) OR KP=((best OR code OR good) NEAR/2 (practice OR practices)) | 23947 |
|  | 32 | SO=(method* or policy or policies or systems or services) | 44059 |
|  | 33 | #32 OR #31 OR #30 | 3305697 |
| G+H | 34 | #33 AND #29 | 5741 |
| I. Guideline development Manuals | 35 | TI=((guideline$ OR guidance) NEAR/0 develop* NEAR/3 (method* OR manual$ OR primer$ OR "how to" OR handbook$ OR framework$ OR checklist$ OR hand-book$ OR frame-work$ OR check-list$ OR step$ OR tutorial$ OR "white paper$" OR toolkit$ OR tool-kit$)) OR AK=((guideline$ OR guidance) NEAR/0 develop* NEAR/3 (method* OR manual$ OR primer$ OR "how to" OR handbook$ OR framework$ OR checklist$ OR hand-book$ OR frame-work$ OR check-list$ OR step$ OR tutorial$ OR "white paper$" OR toolkit$ OR tool-kit$)) OR KP=((guideline$ OR guidance) NEAR/0 develop* NEAR/3 (method* OR manual$ OR primer$ OR "how to" OR handbook$ OR framework$ OR checklist$ OR hand-book$ OR frame-work$ OR check-list$ OR step$ OR tutorial$ OR "white paper$" OR toolkit$ OR tool-kit$)) | 80 |
|  | 36 | TI=((guideline$ OR guidance OR recommendations) NEAR/2 (manual OR handbook OR hand-book)) OR AK=((guideline$ OR guidance OR recommendations) NEAR/2 (manual OR handbook OR hand-book)) OR KP=((guideline$ OR guidance OR recommendations) NEAR/2 (manual OR handbook OR hand-book)) | 183 |
|  | 37 | TS=((manual$ OR guidance OR guideline$ OR guide$ OR primer$ OR "how to" OR tutorial$ OR recommendation$ OR handbook$ OR framework$ OR checklist$ OR hand-book$ OR frame-work$ OR check-list$ OR step$ OR "white paper$" OR toolkit$ OR tool-kit$) NEAR/2 (method* OR conduct* OR develop* OR undertak* OR perform* OR prepar* OR produc* OR creat* OR design* OR report*) NEAR/2 ("public health guid*" OR "evidence based guid*" OR "evidence informed guid*" OR "public health recommendations" OR "evidence based recommendations" OR "evidence informed recommendations") ) | 1797 |
|  | 38 | TI=(("evidence based" OR "evidence informed" OR clinical OR practice OR "public health" OR regulatory OR "emergency response" OR ethic* OR implementation) NEAR/0 (guideline$ OR guidance OR recommendation$)) OR AK=(("evidence based" OR "evidence informed" OR clinical OR practice OR "public health" OR regulatory OR "emergency response" OR ethic* OR implementation) NEAR/0 (guideline$ OR guidance OR recommendation$)) OR KP=(("evidence based" OR "evidence informed" OR clinical OR practice OR "public health" OR regulatory OR "emergency response" OR ethic* OR implementation) NEAR/0 (guideline$ OR guidance OR recommendation$)) | 54196 |
|  | 39 | TI=(("evidence based" OR "evidence informed" OR clinical OR practice OR "public health") NEAR/3 ("Horizon scan$" OR "policy brief$" OR "policy formulation$" OR "research agenda$" OR "Global strateg*" OR "Regional strateg*")) OR AK=(("evidence based" OR "evidence informed" OR clinical OR practice OR "public health") NEAR/3 ("Horizon scan$" OR "policy brief$" OR "policy formulation$" OR "research agenda$" OR "Global strateg*" OR "Regional strateg*")) OR KP=(("evidence based" OR "evidence informed" OR clinical OR practice OR "public health") NEAR/3 ("Horizon scan$" OR "policy brief$" OR "policy formulation$" OR "research agenda$" OR "Global strateg*" OR "Regional strateg*")) | 210 |
|  | 40 | TI=(method* OR conduct* OR develop* OR undertak* OR perform* OR prepar* OR produc* OR creat* OR design* OR reporting) OR AK=(method* OR conduct* OR develop* OR undertak* OR perform* OR prepar* OR produc* OR creat* OR design* OR reporting) OR KP=(method* OR conduct* OR develop* OR undertak* OR perform* OR prepar* OR produc* OR creat* OR design* OR reporting) | 11196351 |
|  | 41 | #33 AND (#38 OR #39) AND #40 | 2369 |
| (G+H) or I | 42 | #41 OR #37 OR #36 OR #35 OR #34 | 9451 |
| J. Public Health and healthcare | 43 | WC=(Public, Environmental & Occupational Health OR General & Internal Medicine OR Health Care Sciences & Services OR Health Policy & Services) OR (TS=("public health" OR "evidence based" OR "evidence informed" OR (health* NEAR/2 emergenc*) OR "health systems" OR "health polic*")) OR SO=("public health" OR evidence OR "health systems" OR "health polic*") | 4816918 |
| Part 2: ((G+H) or I)+J | 44 | #43 AND #42 | 4021 |
| Part 1 or 2 | 45 | #44 OR #24 | 8670 |
| Total, since 2000 | 46 | #45 Timespan: 2000-01-01 to 2024-12-31 | 8519 |

LIGHTS (<https://lights.science/>)

24/04/2024

| Filters | Result |
| --- | --- |
| systematic reviews, health technology assessments, meta analyses, clinical practice guidelines | 329 |
| (Results provided by LIGHTS team) | |

# File S3: Input and feedback from methodologists and MAG members

**MAG members**

1. Dr. Zachary Munn,
2. Dr. H.B.M. (Henk) Hilderink,
3. Prof. Rens van de Schoot, and
4. Dr. Keith Ian Quintyne.

**External methodologists**

1. Marcel Mertz
2. Elie Akl
3. Danielle Pollock
4. Nandi Louise Siegfried
5. Karel GM Moons
6. Bianca Albers
7. Jane Noyes
8. Fiona Campbell
